# High-Throughput Analysis of Total Plasma Fatty Acid Composition with Direct *In Situ* Transesterification

**DOI:** 10.1371/journal.pone.0012045

**Published:** 2010-08-09

**Authors:** Claudia Glaser, Hans Demmelmair, Berthold Koletzko

**Affiliations:** Division of Metabolic and Nutritional Medicine, Dr. von Hauner Children's Hospital, University of Munich Medical Center, Munich, Germany; Hospital 12 Octubre Madrid, Spain

## Abstract

**Background:**

Plasma fatty acid (FA) composition reflects dietary intake and endogenous turnover and is associated with health outcomes on a short and long term basis. The total plasma FA pool represents the composition of all FA containing lipid fractions. We developed a simplified and affordable high-throughput method for the analysis of total plasma FA composition, suitable for large studies.

**Methodology/Principal Findings:**

The total lipid FA from 100 µl plasma is transferred in situ into methyl esters, avoiding initial extraction and drying steps. The fatty acid methyl esters are extracted once and analyzed by gas chromatography. For the new direct in situ transesterification method optimal, reaction parameters were determined. Intra-assay analysis (n = 8) revealed coefficients of variation below 4% for FA contributing more than 1% to total FA.

**Conclusions/Significance:**

The results show good agreement with FA concentrations obtained by a reference method. The new direct in situ transesterification method is robust and simple. Sample preparation time and analysis costs are reduced to a minimum. This method is an economically and ecologically superior alternative to conventional methods for assessing plasma FA status in large studies.

## Introduction

Tissue availability of polyunsaturated fatty acids, which depends on both diet and metabolic turnover, has a major impact on human health [1]–[3]. An adequate supply is very important in every stage of life, but particularly for the fetus and neonate to enable optimal visual and cognitive development [4], [5].

Analysis of fatty acid (FA) composition in different blood and lipid fractions seems to be a valuable biomarker to assess the FA status in humans [6]–[8]. Depending on the scientific question, the FA composition can be determined in adipose tissue, erythrocytes, plasma, platelets, whole blood, and other cells or tissues.

The most convenient way to asses FA composition is in whole blood, because separation of plasma and lipid fractions is not required and dried blood spots have been shown as suitable for analysis [9], [10]. However, the procedure is not yet well established [9], [11], [12] and data interpretation seems more difficult, because different influencing factors have to be considered. A crucial influencing factor is the hematocrit, which depends on gender [13] and age, e.g. hematocrit is higher in neonates than adults and decreases during the first months of life [14]. The hematocrit may be altered by factors such as hypertension [13], pulmonary and cardiac diseases, and pregnancy [15]. Variation of hematocrit may lead to misinterpretation of whole blood FA data because the FA composition of plasma and red blood cells, the main components of whole blood, differs significantly [6].

Analytical precision can be improved by analyzing the FA composition in plasma glycerophospholipids, which contain high percentages of e.g. docosahexaenoic acid and hence are a sensitive biomarker for long chain polyunsaturated FA body status [6]. The analysis of plasma glycerophospholipid FA composition can be performed with a high-throughput methodology [16] in large studies. In addition to this very sensitive method focusing on a specific plasma lipid fraction, it could be revealing to analyze the plasma total FA pool, which represents a mixture of all plasma lipid fractions that contain FA moieties, in particular cholesteryl esters, nonesterified FA, phospholipids, and triglycerides. The FA composition is typical for each lipid class, as specific FA are preferentially partitioned into different plasma lipid pools [17]. Thus, analysis of the total FA pool offers the opportunity to determine overall changes in plasma FA status. However, to assess total FA composition in large studies, methods have to be robust and affordable.

Lepage and Roy [18] developed the first direct transesterification method to assess total FA composition in plasma. Masood, Stark and Salem [19] presented in 2005 a simplified version of the original method of Lepage and Roy. In 2008 Masood and Salem [20] published a modified version of this method, which enables a half automated sample preparation.

We aimed to simplify the assessment of total FA composition in plasma with as few sample preparation steps as possible to enable its application in large studies. Furthermore, we tried to confine sample volume, consumable, reagent, and solvent requirements to a minimum and perform all preparation steps in one vial. We compared our method with a reference method and examined the reliability and limitations of the new method.

## Materials and Methods

### Reagents and biological material

We used anonymous leftover plasma samples that were originally obtained from patients of the Dr. von Hauner Children's Hospital for clinical diagnostics. Five anonymous patient samples were pooled. This pooled sample was aliquoted, stored, and used for analysis of intra-assay reproducibility and the influence of different reaction conditions. Another five anonymous patient samples were pooled to obtain the sample used for storability analysis. For each of the sixteen samples used for comparison analysis of the new with the reference method, five anonymous patient samples were pooled. The ethical committee of the University of Munich Medical Faculty approved this procedure and approved that no informed consent was needed in this case.

Analytical-grade chloroform, hexane, methanol, and water were purchased from Merck KGaA (Darmstadt, Germany) and methanolic HCl (3 N) was obtained from Sigma-Aldrich (Taufkirchen, Germany). Pentadecanoic acid, cholesteryl pentadecanoate, tripentadecanoin, and 1,2-dipentadecanoyl-sn-glycero-3-phosphocholine (Sigma-Aldrich) were dissolved in methanol/chloroform (35∶15) and used as internal standard. To prevent FA oxidation 2 g/l 2,6-di-*tert*-butyl-*p*-cresol (Sigma-Aldrich) was added to the internal standard. The external standard (GLC-85), containing 32 fatty acid methyl esters (FAME), was purchased from Nu-Check Prep, Inc. (Elysian, MN, USA). A mixture of sodium carbonate, sodium hydrogen carbonate and sodium sulfate (1∶2∶2, Merck KGaA) was applied as buffer for neutralization after acid catalyzed transesterification.

### New direct in situ transesterification method

**Figure 1:**
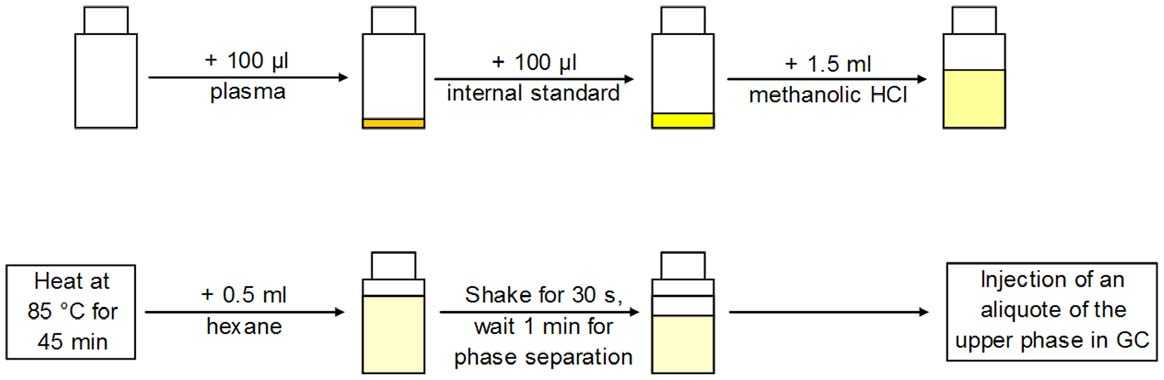
Schematic diagram of the procedure according the direct in situ transesterification method to assess the total fatty acid composition in a plasma sample (GC, gas chromatography).

We developed a method for the analysis of total plasma FA compositions requiring small plasma (100 µl), reagent (1.5 ml), and solvent volumes (0.6 ml) and few sample preparation steps. According to the newly developed method, 100 µl of plasma, 100 µl of internal standard and 1.5 ml methanolic HCl (3 N, containing 2 g/l 2,6-di-*tert*-butyl-*p*-cresol) were combined in closed glass tubes. The samples were shaken for 30 s and heated to 85°C for 45 min. After cooling to room temperature, 0.5 ml hexane (containing 2 g/l 2,6-di-*tert*-butyl-*p*-cresol) was added and the tubes were shaken for 30 s. Phase separation occurred after approx. 1 min at room temperature. For storage and gas chromatographic (GC) analysis an aliquot of the upper hexane phase was transferred into 2 ml vials. A schematic diagram of the procedure is given in Figure 1.

### Reference method

We analyzed the total plasma FA composition according to a reference method to compare the results with that obtained by the new method. For this purpose, we used the standard procedure established in our laboratory, which is based on Folch extraction. In detail, 100 µl of internal standard was added to 250 µl plasma and lipids were extracted by a modified Folch method [21] using chloroform/methanol (2∶1, v/v). The extract was washed two times with NaCl solution (2% in water) and subsequently dried at 30°C under reduced pressure. For methyl ester synthesis the extract was taken up in 400 µl chloroform/methanol and 1.5 ml methanolic HCl (3 N) were added. The tubes were closed, shaken for 30 s, and heated to 85°C for 45 min. Samples were neutralized with carbonate buffer after cooling to room temperature. 1 ml hexane was added for FAME extraction. After centrifugation at 900×g for 5 min the upper hexane phase was transferred into a further glass tube and the extraction was repeated. The extracts were combined, taken to dryness under nitrogen flow at room temperature, and taken up in 50 µl hexane (containing 2 g/l 2,6-di-*tert*-butyl-*p*-cresol) for GC analysis.

### Chromatography

The individual FAME were quantified by GC with flame ionization detection. GC analysis was performed on an Agilent 5890 series II GC (Agilent, Waldbronn, Germany) using a BPX 70 column (25 m×0.22 mm, 25 µm film, SGE, Weiterstadt, Germany). The temperature program started with an initial temperature of 150°C, which was increased with 2.5°C/min to 180°C and 1.5°C/min to 200°C followed by an 1 min isothermal period. Helium was used as carrier gas, starting with a column head pressure of 0.9 bar, which was increased with 0.02 bar/min to 1.2 bar, with 0.05 bar/min to 1.5 bar, and 0.1 bar/min to the final pressure of 2.0 bar.

### Data quantitation

Peak integration was performed using EZChrom Elite version 3.1.7. Individual FAME were identified using authentic standards for comparisons. We used a FAME mixture (GLC-85) as external standard, which was analyzed directly by GC and used to determine the response of each FAME relative to pentadecanoic acid methyl ester.

### Method comparison and statistical analysis

Results were expressed as absolute plasma concentrations (mg/l) and as percentage values (% wt/wt), and FA data were presented as mean. Intra-assay reproducibility was obtained by analyzing 8 aliquots of a pooled plasma sample at an interval of 1 week. Coefficients of variation (CV, %) were used to express analytical precision. The statistical differences between both methods were obtained by analyzing 16 different plasma samples (pooled) and paired t-test was used for comparison between the mean values. All statistical analyses were performed with SPSS for Windows, Version 15.0.1 (SPSS Inc., Chicago, IL, USA).

## Results

Intra-assay reproducibility obtained by the analysis of 8 aliquots of one plasma sample at an interval of 1 week was determined for both methods. Coefficients of variation (Table 1) for all FA contributing more than 1% of total FA were below 4% with the direct in situ transesterification method. Analysis of C22:0, contributing 0.34% of total FA, showed the highest CV (8%).

**Table 1:** Intra-assay *(8 independent measurements of 1 sample)* reproducibility of total lipid fatty acid (FA) concentrations (mg/l) and compositions (% wt/wt) obtained by analysis according to the reference and the direct in situ transesterification method (CV, coefficient of variation; PUFA, polyunsaturated fatty acid).

*FA*	*Reference method*				*Direct in situ transesterification method*			
	FA concentration		FA composition		FA concentration		FA composition	
	Mean	CV	Mean	CV	Mean	CV	Mean	CV
Saturated FA								
C14:0	51.90	3.0	1.53	3.5	70.90	3.7	1.87	3.6
C16:0	901.81	2.6	26.54	1.2	975.65	1.7	25.76	0.7
C17:0	11.48	2.9	0.34	2.3	11.55	3.9	0.30	3.2
C18:0	276.75	5.9	8.14	4.2	278.15	1.5	7.34	0.7
C20:0	10.39	3.4	0.31	1.8	8.60	4.3	0.23	4.1
C22:0	21.54	2.9	0.63	2.2	16.15	7.9	0.43	8.0
C24:0	17.53	4.4	0.52	4.2	15.10	7.0	0.40	7.4
Monounsaturated FA								
C14:1	3.99	7.9	0.12	8.7	6.45	4.0	0.17	3.7
C16:1n-7	93.56	1.4	2.75	1.3	107.40	3.1	2.84	2.9
C18:1n-7	65.49	2.1	1.93	0.9	69.40	3.2	1.83	2.9
C18:1n-9	839.26	2.1	24.70	0.9	914.55	1.3	24.14	0.3
C20:1n-9	7.31	3.5	0.22	3.1	7.10	5.8	0.19	5.7
C24:1n-9	31.16	3.5	0.92	3.1	31.35	5.7	0.83	5.4
n-9 PUFA								
C20:3n-9	4.48	2.6	0.13	0.8	5.90	4.8	0.16	4.9
n-6 PUFA								
C18:2n-6	719.04	1.8	21.16	0.7	836.60	1.4	22.09	0.3
C18:3n-6	14.91	6.0	0.44	7.0	18.00	2.4	0.48	1.4
C20:2n-6	6.01	4.4	0.18	5.0	6.40	5.8	0.17	5.2
C20:3n-6	47.86	2.4	1.41	1.5	58.50	1.7	1.54	1.1
C20:4n-6	172.53	2.3	5.08	1.7	219.50	1.1	5.79	1.0
C22:4n-6	6.78	4.7	0.20	5.2	8.50	7.0	0.22	7.0
C22:5n-6	4.74	9.5	0.14	10.1	6.50	7.2	0.17	7.8
n-3 PUFA								
C18:3n-3	13.83	1.9	0.41	1.2	17.20	1.8	0.45	1.2
C20:5n-3	18.41	9.5	0.54	9.6	21.55	2.9	0.57	2.2
C22:5n-3	13.38	3.6	0.39	3.4	17.15	3.4	0.45	3.0
C22:6n-3	43.78	2.8	1.29	2.8	59.80	1.9	1.58	1.6
Total FA								
	3397.89	2.1			3787.95	1.3		

Furthermore, we analyzed the FA concentrations of sixteen different pooled plasma samples (Table S1). The measured total FA concentrations for these samples were 2715.9 mg/l (range 1851.1 mg/l–5409.8 mg/l) with the reference method and 2913.2 mg/l (range 1936.0 mg/l–5865.0 mg/l) with the direct in situ transesterification method. Absolute plasma concentrations (mg/l) were significantly higher for most of the analyzed FA with the direct in situ transesterification method compared to the reference method. There were no significant differences in the concentrations for the saturated FA C17:0, C20:0, and C22:0, for the monounsaturated FA C14:1n-5 and C20:1n-9, and for the polyunsaturated FA C20:2n-6, C22:4n-6, C22:5n-6, C18:3n-3, and C20:5n-3. The concentration of C18:3n-6 was higher obtained with the reference methods compared to the direct in situ transesterification method. For all other FA, particularly docosahexaenoic acid, absolute plasma concentrations were higher with the direct in situ transesterification method than with the reference method. However, both methods revealed very similar percentage FA values (% wt/wt).

We assessed the effects of reaction conditions on the determined FA in more detail (Table S2). Both, longer reaction times (90 min instead of 45 min) as well as an increase of the reaction temperature from 85°C to 100°C led to higher concentration yields for C20:0, C22:0, C24:0, and C24:1n-9 (up to 40%). No further increase in concentrations was obtained with prolonging reaction times beyond 90 min. However, the effect on percentage FA compositions is very small, because of the low abundance of these four FA (<1% of total FA).

Storage of the GC ready derivatives for one month at −20°C did not lead to appreciable alterations of FA concentrations (Table 2).

**Table 2:** Fatty acid (FA) values obtained with the new direct in situ transesterification method for a plasma sample analyzed by gas chromatography directly after sample preparation, then stored for 1 month at −20°C and analyzed again (PUFA, polyunsaturated fatty acid).

*FA*	*FA concentration (mg/l)*				FA composition (% wt/wt)			
	Start	4 weeks	Difference		Start	4 weeks	Difference	
			mg/l	%			% wt/wt	%
Saturated FA								
C14:0	57.40	57.60	0.20	0.3	1.66	1.66	0.00	0.1
C16:0	896.20	899.20	3.00	0.3	25.86	25.88	0.02	0.1
C17:0	10.80	10.80	0.00	0.0	0.31	0.31	0.00	−0.3
C18:0	258.40	263.40	5.00	1.9	7.46	7.58	0.13	1.7
C20:0	8.10	8.40	0.30	3.7	0.23	0.24	0.01	3.4
C22:0	14.80	15.60	0.80	5.4	0.43	0.45	0.02	5.1
C24:0	13.90	14.80	0.90	6.5	0.40	0.43	0.02	6.2
Monounsaturated FA								
C14:1n-5	5.40	5.40	0.00	0.0	0.16	0.16	0.00	−0.3
C16:1n-7	99.60	97.80	−1.80	−1.8	2.87	2.81	−0.06	−2.1
C18:1n-7	65.80	67.20	1.40	2.1	1.90	1.93	0.04	1.9
C18:1n-9	860.80	857.00	−3.80	−0.4	24.84	24.66	−0.17	−0.7
C20:1n-9	8.20	7.80	−0.40	−4.9	0.24	0.22	−0.01	−5.1
C24:1n-9	27.20	29.40	2.20	8.1	0.78	0.85	0.06	7.8
n-9 PUFA								
C20:3n-9	5.00	5.20	0.20	4.0	0.14	0.15	0.01	3.7
n-6 PUFA								
C18:2n-6	762.60	758.00	−4.60	−0.6	22.00	21.82	−0.19	−0.9
C18:3n-6	15.60	15.40	−0.20	−1.3	0.45	0.44	−0.01	−1.5
C20:2n-6	6.00	6.20	0.20	3.3	0.17	0.18	0.01	3.1
C20:3n-6	52.60	52.60	0.00	0.0	1.52	1.51	0.00	−0.3
C20:4n-6	187.80	192.00	4.20	2.2	5.42	5.53	0.11	2.0
C22:4n-6	7.60	7.40	−0.20	−2.6	0.22	0.21	−0.01	−2.9
C22:5n-6	4.50	4.80	0.30	6.7	0.13	0.14	0.01	6.4
n-3 PUFA								
C18:3n-3	15.00	14.60	−0.40	−2.7	0.43	0.42	−0.01	−2.9
C20:5n-3	16.60	17.20	0.60	3.6	0.48	0.50	0.02	3.4
C22:5n-3	16.00	15.60	−0.40	−2.5	0.46	0.45	−0.01	−2.7
C22:6n-3	50.00	51.20	1.20	2.4	1.44	1.47	0.03	2.1
Total FA								
	3465.90	3474.60	8.70	0.3				

## Discussion

The new analysis method allows for rapid, precise, and reproducible analysis of total plasma FA. Significantly higher absolute plasma concentrations for most of the analyzed FA were obtained by direct in situ transesterification compared to our reference method, presumably because losses due to non-quantitative extraction are avoided by the direct in situ transesterification method as previously noted by other authors [18], [22]. Of importance, the addition of an internal standard dissolved in an organic solvent to the plasma sample obviously is not fully adequate for the extraction of lipoprotein bound lipids.

Absolute plasma FA concentration of C20:0 obtained by direct in situ transesterification for 45 min at 85°C was lower than concentrations found with the reference method. Longer reaction times and/or higher temperatures enabled more complete direct in situ transesterification of C20:0, C22:0, C24:0, and C24:1n-9. These FA are mainly found in the phospholipid fraction of human plasma [23]. The concentrations of these FA in glycerophospholipids are below the quantification level, whereas they show a high abundance in sphingomyelin [23]. Masood et al. [19] obtained a decline of 40% of certain FA (C20:0, C22:0, C24:0 and C24:1n-9) with transesterification at 75°C for 60 min–90 min with their open-tube method compared to the direct transesterification method of Lepage and Roy [18]. They considered that more extreme conditions such as higher temperatures and longer heating times were required for complete transesterification of sphingomyelin FA, because of their less reactive amide bonds. Quantitative transesterification of these FA would require at least duplication of reaction time. However, the benefit of exact determination of the concentrations of C20:0, C22:0, and C24:0 seems small for most clinical and research applications, which must be weighed against the loss of productivity and the increasing risk of FA degradation or isomerization during derivatization [24]–[26]. Moreover, for most applications the percentage FA compositions are of interest, which are constant at the different reaction conditions, because the amounts of C20:0, C22:0, and C24:0 are too small to alter the entire composition.

Different lipoproteins contain distinct amounts of cholesteryl esters, phospholipids, and triglycerides, which have an individual FA composition. Therefore, the FA composition obtained by direct in situ transesterification in plasma reflects the total plasma FA composition given by the composition of different lipoproteins and the non-esterified FA.

In conclusion, the newly developed direct in situ transesterification method presented here, enables analysis of total plasma FA concentrations and percentage contributions from only 100 µl of plasma. The method is robust and simple, because lipid extraction and washing steps as well as neutralization, centrifugation and drying steps are avoided and only one FAME extraction step is required. Sample preparation can be performed in one vial, preparation time is reduced to a minimum, and only small volumes of reagents and solvents are necessary. Thus, the method is economically and ecologically superior to conventional methods, and it is well suitable for the application in large clinical trials and epidemiological studies.

## Supporting Information

Table S1 Differences between the reference and the direct in situ transesterification method for total lipid fatty acid concentrations (mean, mg/l) and compositions (mean, % wt/wt) obtained by the analysis of 16 different plasma samples (pooled).
(0.10 MB DOC)

Table S2 Influence of different reaction conditions on the determined fatty acid (FA) concentrations (mg/l) and compositions (%) (PUFA, polyunsaturated fatty acid).
(0.10 MB DOC)
