# Supplementary material for: High-Throughput Analysis of Total Plasma Fatty Acid Composition with Direct In Situ Transesterification
Source: PLoS One. 2010 Aug 9;5(8):e12045. doi: 10.1371/journal.pone.0012045 (PMC2918509; doi:10.1371/journal.pone.0012045)
Supplement: Table S1 — Differences between the reference and the direct in situ transesterification method for total lipid fatty acid concentrations (mean, mg/l) and compositions (mean, % wt/wt) obtained by the analysis of 16 different plasma samples (pooled). (0.10 MB DOC) [file pone.0012045.s001.doc]

| *FA* | *Reference method* | | *Direct in situ transesterification method* | | *Difference* | | | | | |
| --- | --- | --- | --- | --- | --- | --- | --- | --- | --- | --- |
|  | *FA concentration* | *FA composition* | *FA concentration* | *FA composition* | *FA concentration* | | | *FA composition* | | |
|  |  |  |  |  | *Absolute* | *Percentage* | *Significance* | *Absolute* | *Percentage* | *Significance* |
| Saturated FA |  |  |  |  |  |  |  |  |  |  |
| C14:0 | 36.7 | 1.3 | 40.0 | 1.3 | 3.31 | 9.0 | <0.001 | 0.02 | 1.8 | 0.098 |
| C16:0 | 658.5 | 23.7 | 710.6 | 23.9 | 52.02 | 7.9 | <0.001 | 0.15 | 0.6 | 0.062 |
| C17:0 | 9.0 | 0.3 | 9.2 | 0.3 | 0.27 | 3.0 | 0.171 | -0.01 | -4.1 | 0.866 |
| C18:0 | 212.8 | 8.0 | 233.1 | 8.2 | 20.33 | 9.6 | <0.001 | 0.18 | 2.3 | 0.007 |
| C20:0 | 8.0 | 0.3 | 7.8 | 0.3 | -0.23 | -2.9 | 0.288 | -0.03 | -10.2 | <0.001 |
| C22:0 | 16.6 | 0.6 | 17.4 | 0.6 | 0.82 | 4.9 | 0.172 | -0.01 | -1.9 | 0.375 |
| C24:0 | 14.3 | 0.5 | 16.1 | 0.6 | 1.80 | 12.7 | 0.002 | 0.03 | 5.2 | 0.045 |
| Monounsaturated FA |  |  |  |  |  |  |  |  |  |  |
| C14:1n-5 | 2.6 | 0.1 | 2.6 | 0.1 | 0.04 | 1.7 | 0.756 | -0.004 | -4.7 | 0.278 |
| C16:1n-7 | 72.3 | 2.6 | 75.4 | 2.5 | 3.17 | 4.4 | <0.001 | -0.07 | -2.7 | 0.001 |
| C18:1n-7 | 52.1 | 1.9 | 56.2 | 1.9 | 4.05 | 7.8 | <0.001 | 0.01 | 0.3 | 0.360 |
| C18:1n-9 | 612.7 | 22.1 | 667.8 | 22.5 | 55.06 | 9.0 | <0.001 | 0.38 | 1.7 | <0.001 |
| C20:1n-9 | 5.4 | 0.2 | 5.5 | 0.2 | 0.09 | 1.7 | 0.479 | -0.01 | -5.9 | 0.002 |
| C24:1n-9 | 28.5 | 1.1 | 31.6 | 1.1 | 3.06 | 10.7 | 0.001 | 0.03 | 3.0 | 0.036 |
| n-9 PUFA |  |  |  |  |  |  |  |  |  |  |
| C20:3n-9 | 5.4 | 0.2 | 6.1 | 0.2 | 0.71 | 13.2 | <0.001 | 0.01 | 6.3 | <0.001 |
| n-6 PUFA |  |  |  |  |  |  |  |  |  |  |
| C18:2n-6 | 632.8 | 23.6 | 669.5 | 23.3 | 36.74 | 5.8 | <0.001 | -0.32 | -1.4 | 0.001 |
| C18:3n-6 | 12.3 | 0.4 | 11.8 | 0.4 | -0.51 | -4.2 | 0.012 | -0.05 | -10.9 | <0.001 |
| C20:2n-6 | 5.4 | 0.2 | 5.6 | 0.2 | 0.14 | 2.5 | 0.375 | -0.01 | -5.5 | 0.034 |
| C20:3n-6 | 42.5 | 1.6 | 46.1 | 1.6 | 3.63 | 8.6 | <0.001 | 0.02 | 1.0 | 0.017 |
| C20:4n-6 | 185.8 | 7.1 | 196.7 | 7.0 | 10.87 | 5.8 | <0.001 | -0.11 | -1.5 | 0.014 |
| C22:4n-6 | 7.7 | 0.3 | 7.9 | 0.3 | 0.25 | 3.2 | 0.076 | -0.01 | -3.7 | 0.030 |
| C22:5n-6 | 7.4 | 0.3 | 7.7 | 0.3 | 0.24 | 3.3 | 0.103 | -0.01 | -3.6 | 0.022 |
| n-3 PUFA |  |  |  |  |  |  |  |  |  |  |
| C18:3n-3 | 15.2 | 0.6 | 15.3 | 0.5 | 0.15 | 1.0 | 0.329 | -0.04 | -6.5 | <0.001 |
| C20:5n-3 | 14.2 | 0.5 | 13.7 | 0.5 | -0.51 | -3.6 | 0.067 | -0.05 | -10.2 | <0.001 |
| C22:5n-3 | 13.5 | 0.5 | 13.9 | 0.5 | 0.42 | 3.1 | 0.036 | -0.02 | -4.2 | 0.002 |
| C22:6n-3 | 44.3 | 1.7 | 45.7 | 1.6 | 1.41 | 3.2 | 0.018 | -0.07 | -4.1 | <0.001 |
| Total FA |  |  |  |  |  |  |  |  |  |  |
|  | 2715.9 | - | 2913.2 | - | 197.34 | 7.3 | <0.001 | - | - | - |
