# Supplementary material for: High-Throughput Analysis of Total Plasma Fatty Acid Composition with Direct In Situ Transesterification
Source: PLoS One. 2010 Aug 9;5(8):e12045. doi: 10.1371/journal.pone.0012045 (PMC2918509; doi:10.1371/journal.pone.0012045)
Supplement: Table S2 — Influence of different reaction conditions on the determined fatty acid (FA) concentrations (mg/l) and compositions (%) (PUFA, polyunsaturated fatty acid). (0.10 MB DOC) [file pone.0012045.s002.doc]

| *FA* | *Concentrations (mg/l)* | | | | | | *Compositions (%)* | | | | | |
| --- | --- | --- | --- | --- | --- | --- | --- | --- | --- | --- | --- | --- |
|  | *Transesterification at 85 °C* | | | *Transesterification at 100 °C* | | | *Tranesterification at 85 °C* | | | *Transesterification at 100 °C* | | |
|  | *45 min* | *60 min* | *90 min* | *45 min* | *60 min* | *90min* | *45 min* | *60 min* | *90 min* | *45 min* | *60 min* | *90 min* |
| Saturated FA |  |  |  |  |  |  |  |  |  |  |  |  |
| C14:0 | 68.4 | 73.2 | 66.0 | 68.0 | 67.6 | 66.8 | 1.9 | 2.0 | 1.9 | 1.9 | 1.9 | 1.9 |
| C16:0 | 959.6 | 977.2 | 934.0 | 966.0 | 958.0 | 964.0 | 26.8 | 27.0 | 26.8 | 26.8 | 26.9 | 26.9 |
| C17:0 | 11.2 | 11.6 | 11.2 | 11.2 | 11.6 | 11.6 | 0.3 | 0.3 | 0.3 | 0.3 | 0.3 | 0.3 |
| C18:0 | 276.8 | 276.8 | 268.8 | 276.4 | 273.2 | 276.0 | 7.7 | 7.6 | 7.7 | 7.7 | 7.7 | 7.7 |
| C20:0 | 7.6 | 7.6 | 8.4 | 9.6 | 8.8 | 9.2 | 0.2 | 0.2 | 0.2 | 0.3 | 0.2 | 0.3 |
| C22:0 | 13.6 | 14.0 | 16.0 | 16.8 | 17.2 | 18.0 | 0.4 | 0.4 | 0.5 | 0.5 | 0.5 | 0.5 |
| C24:0 | 12.4 | 16.0 | 17.2 | 17.2 | 17.2 | 18.4 | 0.3 | 0.4 | 0.5 | 0.5 | 0.5 | 0.5 |
| Monounsaturated FA |  |  |  |  |  |  |  |  |  |  |  |  |
| C14:1n-5 | 4.0 | 4.0 | 4.0 | 4.0 | 3.6 | 4.0 | 0.1 | 0.1 | 0.1 | 0.1 | 0.1 | 0.1 |
| C16:1n-7 | 107.6 | 109.2 | 103.2 | 106.8 | 105.6 | 105.2 | 3.0 | 3.0 | 3.0 | 3.0 | 3.0 | 2.9 |
| C18:1n-7 | 68.0 | 70.4 | 67.6 | 69.6 | 69.2 | 69.2 | 1.9 | 1.9 | 1.9 | 1.9 | 1.9 | 1.9 |
| C18:1n-9 | 852.8 | 866.8 | 837.6 | 861.2 | 854.0 | 860.0 | 23.8 | 23.9 | 24.1 | 23.9 | 23.9 | 24.0 |
| C20:1n-9 | 6.8 | 6.8 | 6.8 | 6.8 | 6.8 | 7.2 | 0.2 | 0.2 | 0.2 | 0.2 | 0.2 | 0.2 |
| C24:1n-9 | 25.6 | 28.4 | 32.4 | 34.4 | 35.6 | 36.4 | 0.7 | 0.8 | 0.9 | 1.0 | 1.0 | 1.0 |
| n-9 PUFA |  |  |  |  |  |  |  |  |  |  |  |  |
| C20:3n-9 | 6.0 | 5.6 | 5.2 | 5.2 | 5.6 | 5.2 | 0.2 | 0.2 | 0.1 | 0.1 | 0.2 | 0.1 |
| n-6 PUFA |  |  |  |  |  |  |  |  |  |  |  |  |
| C18:2n-6 | 771.2 | 777.6 | 740.4 | 769.6 | 760.4 | 763.6 | 21.6 | 21.5 | 21.3 | 21.4 | 21.3 | 21.3 |
| C18:3n-6 | 16.8 | 16.8 | 15.6 | 16.8 | 16.4 | 16.4 | 0.5 | 0.5 | 0.4 | 0.5 | 0.5 | 0.5 |
| C20:2n-6 | 6.0 | 5.6 | 5.6 | 5.6 | 5.6 | 5.6 | 0.2 | 0.2 | 0.2 | 0.2 | 0.2 | 0.2 |
| C20:3n-6 | 52.4 | 51.6 | 49.6 | 51.6 | 50.4 | 51.2 | 1.5 | 1.4 | 1.4 | 1.4 | 1.4 | 1.4 |
| C20:4n-6 | 194.0 | 191.2 | 184.0 | 191.6 | 188.8 | 189.2 | 5.4 | 5.3 | 5.3 | 5.3 | 5.3 | 5.3 |
| C22:4n-6 | 6.8 | 6.8 | 7.2 | 6.8 | 7.2 | 6.8 | 0.2 | 0.2 | 0.2 | 0.2 | 0.2 | 0.2 |
| C22:5n-6 | 6.4 | 6.4 | 4.8 | 6.0 | 6.4 | 5.2 | 0.2 | 0.2 | 0.1 | 0.2 | 0.2 | 0.1 |
| n-3 PUFA |  |  |  |  |  |  |  |  |  |  |  |  |
| C18:3n-3 | 15.6 | 15.6 | 14.8 | 15.6 | 15.6 | 15.2 | 0.4 | 0.4 | 0.4 | 0.4 | 0.4 | 0.4 |
| C20:5n-3 | 18.4 | 17.2 | 16.8 | 17.6 | 17.6 | 16.8 | 0.5 | 0.5 | 0.5 | 0.5 | 0.5 | 0.5 |
| C22:5n-3 | 17.0 | 17.0 | 14.4 | 14.4 | 14.8 | 15.2 | 0.5 | 0.5 | 0.4 | 0.4 | 0.4 | 0.4 |
| C22:6n-3 | 52.0 | 50.8 | 48.0 | 50.8 | 49.6 | 50.0 | 1.5 | 1.4 | 1.4 | 1.4 | 1.4 | 1.4 |
| Total FA |  |  |  |  |  |  |  |  |  |  |  |  |
|  | 3577.0 | 3624.2 | 3479.6 | 3599.6 | 3566.8 | 3586.4 |  |  |  |  |  |  |
